# Stepwise Ethanol-Water Fractionation of Enzymatic Hydrolysis Lignin to Improve Its Performance as a Cationic Dye Adsorbent

**DOI:** 10.3390/molecules25112603

**Published:** 2020-06-03

**Authors:** Wenjie Sui, Tairan Pang, Guanhua Wang, Cuiyun Liu, Ashak Mahmud Parvez, Chuanling Si, Chao Li

**Affiliations:** 1 State Key Laboratory of Food Nutrition and Safety, College of Food Science and Engineering, Tianjin University of Science & Technology, Tianjin 300457, China; 2 Tianjin Key Laboratory of Pulp and Paper, College of Light Industry Science and Engineering, Tianjin University of Science and Technology, Tianjin 300457, China; pangtr1995@163.com (T.P.); zclcy1214@163.com (C.L.); sichli@tust.edu.cn (C.S.); 3 Hunan BISEN Environmental & Energy Co., Ltd., Changsha 410100, China; chaoli_tu@tsinghua.edu.cn; 4 Department of Mechanical Engineering, University of New Brunswick, Fredericton, NB E3B 5A3, Canada; Ashak.Parvez@unb.ca; 5 School of Environment, Tsinghua University, Beijing 100084, China

**Keywords:** enzymatic hydrolysis lignin, sequential dissolution fractionation, methylene blue adsorption capacity

## Abstract

In this work, lignin fractionation is proposed as an effective approach to reduce the heterogeneity of lignin and improve the adsorption and recycle performances of lignin as a cationic dye adsorbent. By stepwise dissolution of enzymatic hydrolysis lignin in 95% and 80% ethanol solutions, three lignin subdivisions (95% ethanol-soluble subdivision, 80% ethanol-soluble subdivision, and 80% ethanol-insoluble subdivision) were obtained. The three lignin subdivisions were characterized by gel permeation chromatography (GPC), FTIR, 2D-NMR and scanning electron microscopy (SEM), and their adsorption capacities for methylene blue were compared. The results showed that the 80% ethanol-insoluble subdivision exhibited the highest adsorption capacity and its value (396.85 mg/g) was over 0.4 times higher than that of the unfractionated lignin (281.54 mg/g). The increased adsorption capacity was caused by the enhancement of both specific surface area and negative Zeta potential. The maximum monolayer adsorption capacity of 80% ethanol-insoluble subdivision by adsorption kinetics and isotherm studies was found to be 431.1 mg/g, which was much higher than most of reported lignin-based adsorbents. Moreover, the 80% ethanol-insoluble subdivision had much higher regeneration yield (over 90% after 5 recycles) compared with the other two subdivisions. Consequently, the proposed fractionation method is proved to be a novel and efficient non-chemical modification approach that significantly improves adsorption capacity and recyclability of lignin.

## 1. Introduction

Nowadays, synthetic organic dyes are widely applied in various industries, such as printing, paper, textile, electroplating, pulp mill, food and cosmetic productions [1]. During the dyeing process, 10–15% of dye is discharged in the effluent [2,3], which not only creates an aesthetic nuisance to the environment but also increases the overall loading of chemical oxygen demand (COD) in the receiving water. In order to remove dyes from industrial effluents, numerous technologies have been developed, such as adsorption [4,5], coagulation [6], chemical oxidation [7], photocatalytic degradation [8] and biological treatment [2]. Among them, adsorption has been preferred owing to its low operation cost and high efficiency [4,9]. Various kinds of adsorbents have been reported for dyes removing from water effluent, such as activated carbon [10,11], bentonite [12], and synthesized polymers [13]. Currently, there are increasing interests in utilizing biomass derived materials as a dye adsorbent due to their benign properties, such as acceptable specific strength, low cost, no health risk and sustainability [9,14].

Lignin, as the dominant aromatic component of lignocellulosic biomass, has attracted considerable attention in the pollutant adsorption field since it contains a number of functional groups (e.g., phenolic hydroxyl and carboxyl) that serve as potential active sites for adsorption [15,16,17,18,19]. Owing to the negative charge of these functional groups in water environment, unmodified lignin possesses preferable adsorption ability for cationic dyes [16,20,21,22]. Zhang et al. investigated the methylene blue adsorption by organosolv lignin from rice straw and found that the adsorption capacity was 40.02 mg/g at 20 °C, which was comparable with other lignocellulose adsorbents [23]. Menkiti et al. utilized alkaline lignin extracted from elephant grass to remove aqueous crystal violet dye and their results indicated that the crystal violet uptake capacity of lignin was about 25.0 mg/g at 30 °C [24]. Feng et al. analyzed the methylene blue removal behaviors of deacetylated lignin from acetic acid pulping of eucalyptus and suggested that the modification and isolation processes of lignin could improve the adsorption ability from 18.2 to 63.3 mg/g owing to the enhanced contents of phenolic hydroxyl and carboxylic groups [22].

However, lignin is a typically heterogeneous biopolymer and the heterogeneity of lignin leads to its inhomogeneous properties [25], which may also involve the adsorption capacities. Although many efforts have been dedicated to the study of different sources/types of lignin as potential absorbents for removal of cationic dyes from wastewater, until now, no attempts to explore the effect of heterogeneity of one particular lignin sample on its dye adsorption capacity has been reported. It is known that lignin fractionation is a simple but efficient process to decrease the lignin inhomogeneity by subdividing the heterogeneous lignin into different fractions (subdivisions), which show obviously reduced heterogeneity in both molecular weight and chemical structure [26]. Thus, the effect of heterogeneity on lignin adsorption performance can be then investigated by comparing the adsorption performances of the subdivisions obtained from lignin fractionation.

In this work, enzymatic hydrolysis lignin (EHL), a main byproduct from cellulosic ethanol production, was fractionated into three subdivisions by stepwise dissolution in 95% and 80% ethanol solvents. The effect of lignin heterogeneity on its dye adsorption capacity was investigated by a comparison of the methylene blue (MB, a typical cationic dye pollutant) adsorption capacities of the three obtained lignin subdivisions. Interestingly, it was found that the three lignin subdivisions exhibited remarkably different adsorption capacities under the optimized conditions (e.g., pH, adsorbent dosage, temperature and time) and the 80% ethanol-insoluble subdivision had much higher adsorption capacity than the other two subdivisions. Moreover, the specific surface area and the Zeta potential of the three lignin subdivisions were measured to analyze the possible formation mechanism of different adsorption capacities among the three subdivisions. With respect to the recyclability study, the MB loaded lignin adsorbents were regenerated using a common ethanol washing process and afterwards reused for MB removal from a fresh solution.

## 2. Results and Discussion

### 2.1. Lignin Fractionation by Stepwise Dissolution

**Figure 1:**
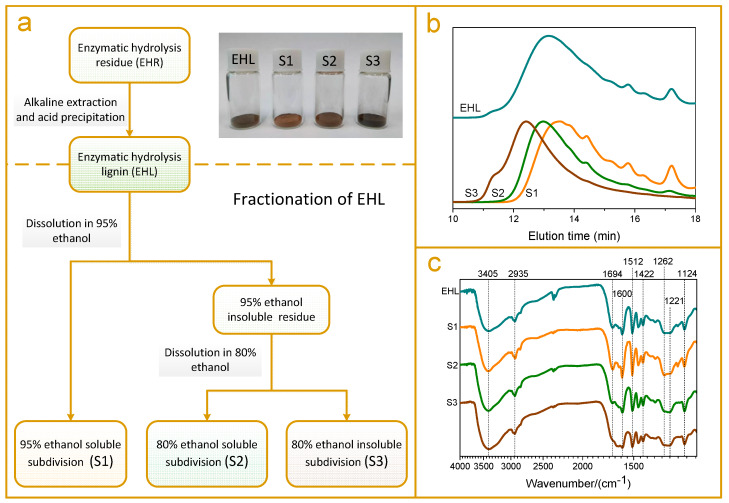
(**a**) Procedure for stepwise ethanol-water fractionation of enzymatic hydrolysis lignin (EHL), inset: pictures of S1, S2, S3, and EHL; (**b**) Molecular weight distributions of S1, S2, S3, and EHL; (**c**) FTIR spectra of S1, S2, S3, and EHL.

By stepwise dissolution in 95% and 80% ethanol solvents, the enzymatic hydrolysis lignin (EHL) was fractionated into three subdivisions (Figure 1a), namely, 95% ethanol soluble subdivision (S1), 80% ethanol soluble subdivision (S2), and 80% ethanol insoluble subdivision (S3). The yields of the three lignin subdivisions were 22.87%, 27.803% and 45.34%, respectively (Table 1). As shown in Figure 1a, the color of the lignin subdivisions was varied, which turned from light brown (S1), to dark brown (S2), and finally to black (S3, Figure 1a). The molecular weight of the three subdivisions increased from S1 to S3 (Figure 1b) and all the three subdivisions exhibited reduced polydispersity compared with the original EHL (Table 1). These results suggest that the fractionation method using sequential dissolution in 95% and 80% ethanol realizes the separation of different molecular weight lignin subdivisions from the heterogeneous EHL. The fractionation rationale lies in the different solubility of lignin subdivisions with different molecular weights in the selective ethanol/water solvents [25].

The FTIR spectra of EHL and its three subdivisions basically conformed to the typical absorption bands of lignin (Figure 1c). The broad band at 3405 cm^−1^ was assigned to the stretching of hydroxyl and the signal at 2935 cm^−1^ was attributed to C-H stretching vibration from methyl and methane [25,27]. Three sharp peaks observed at 1600, 1512 and 1422 cm^−^^1^ were caused by the aromatic skeleton vibrations, indicating the primary structure of lignin [28]. The bands at 1221 and 1124 cm^−1^ reflected syringyl structures in lignin, while the band at 1262 cm^−1^ was associated with guaiacyl units. One obvious difference among the three subdivisions was that the S1 had much higher absorption intensity at 1694 cm^−1^ compared to those noticed in the other two subdivisions. The signal at 1694 cm^−1^ was assigned to the unconjugated β-ketone in the side chain of lignin unit, which was principally formed by the breakage of the β-*O*-4 bond during the steam explosion process [28]. Thus, the results indicate that the depolymerized lignin produced during the steam explosion process is enriched in S1, which agree well with the results from molecular weight analysis.

**Figure 2:**
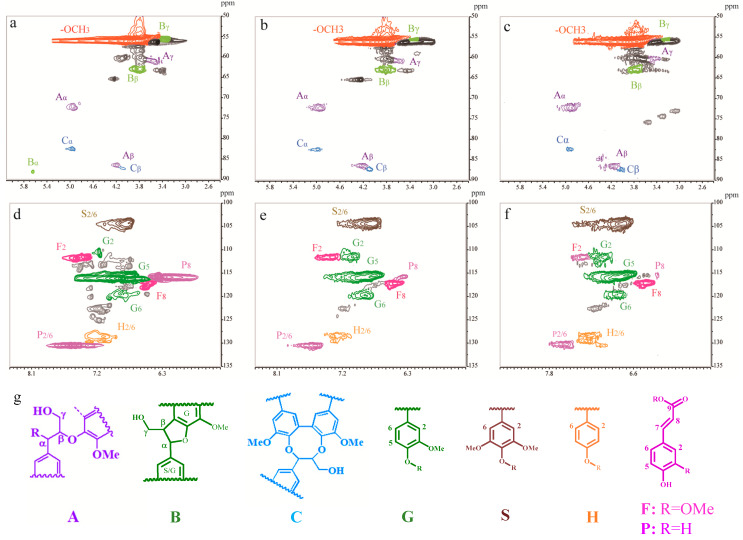
Side-chain (**a−c**) and aromatic regions (**d−f**) of 2D-NMR (HSQC) spectra of three lignin subdivisions (S1: **a** and **d**; S2: **b** and **e**; S3: **c** and **f**). Main substructures identified: (A) β-*O*-4′ aryl ether linkage; (B) phenyl-coumaran structure formed by β-5′ and α-*O*-4′ linkages; (C) dibenzodioxocin structure; (G) guaiacyl unit; (S) syringyl unit; (H) *p*-hydroxyphenyl unit; (F) ferulic acid; (P) *p*-coumaric acid.

2D-NMR analysis was also conducted to acquire a more detailed understanding of the chemical structure of the three lignin subdivisions (Figure 2). In the side chain region (δC/δH 50−90/2.6−5.8), three typical interunit linkages, including β-*O*-4′ structure (A), pinoresinol structure (B), and dibenzodioxocin structure (C), were identified [29,30]. It was found that the signal intensities for aryl ester linkage (β-*O*-4′ structure, A) were stronger for S2 and S3 while the C-C bonding (structure B and C) signals were more noticeable in S1. This phenomena could be explained by the fact that it is relatively easier to break the aryl ester linkage during the steam explosion pretreatment [31,32,33] and the extraction process using NaOH aqueous solution [34]. Thus, the degraded lignin with low-molecular-weight lignin (S1) exhibited a lower amount of β-*O*-4′ aryl ester linkage. The aromatic region in the 2D-HSQC spectra exhibited obvious guaiacyl (G), syringyl (S), and *p*-hydroxyphenyl (H) rings, suggesting that the lignin used in this work was a GSH-type lignin. Additionally, the phenolic acid signals were much more evident in S1 (Figure 2). This result supports the previously reported findings that the phenolic acids are enriched in the low-molecular-weight subdivision since they show good solubility in 95% ethanol solution [25].

**Figure 3:**
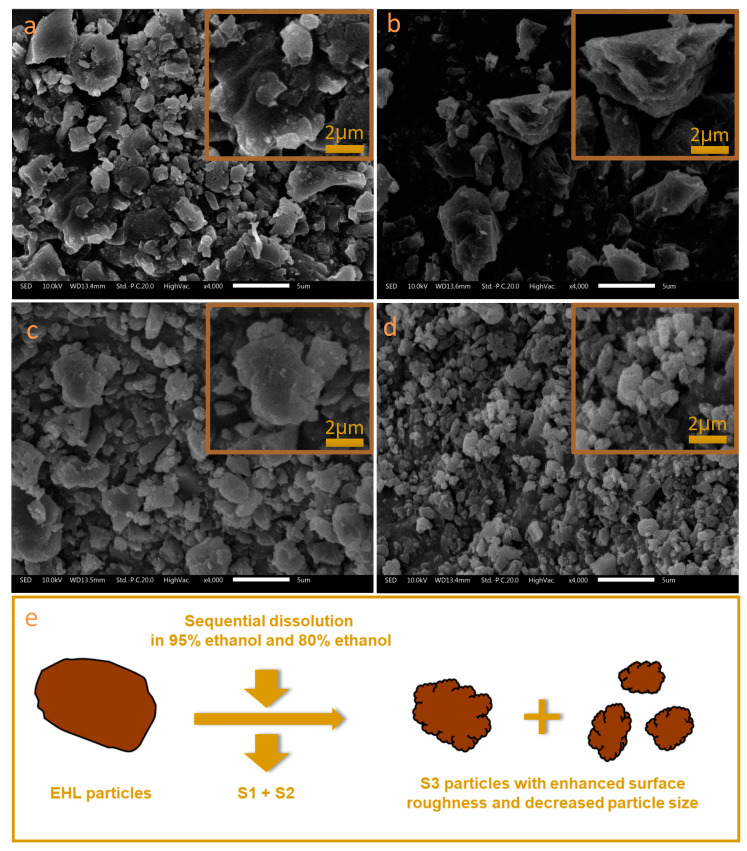
Scanning electron microscopy (SEM) images of EHL (**a**), S1 (**b**), S2 (**c**), and S3 (**d**). The scale bar for the SEM images is 5 μm. Schematic showing morphological changes from EHL particles to the 80% ethanol insoluble subdivision (S3) particles (**e**).

The surface morphology of the three lignin subdivisions as well as the parent EHL is presented in Figure 3. It was observed that the particles of EHL, S1, and S2 were large in size with smooth surface and sharp edges. Nair et al. observed the microcosmic surface of alkaline lignin using SEM and they also found that the alkali lignin particles were larger in size with sharp edges and lesser surface roughness [35]. However, S3 exhibited visibly decreased particle size and enhanced surface roughness. This result can be explained by the preparation processes of the lignin samples. EHL, S1 and S2 were prepared by precipitation from lignin solution using pH adjusting or solvent evaporation (Section 3.2 and Section 3.3). This precipitation processes resulted in the aggregation of small lignin particles and the formation of large lignin particles with a smooth surface. However, the S3 was obtained from EHL after sequential dissolution of EHL in 95% and 80% ethanol solutions. The dissolution of low molecular weight lignin (S1 and S2) resulted in the formation of a porous structure on the lignin particle and even the fragmenting of lignin particles, which increased the surface roughness and decreased the particle size (Figure 3e).

Due to the increase of the surface roughness and the decrease of particle size, the specific surface area of S3 (4.84 m^2^/g) was the highest among the four lignin samples, which increased 67.57% compared with the parent EHL (2.89 m^2^/g, Table 1). The specific surface area of EHL was slightly higher than those of alkali lignin [35] and kraft lignin [36], probably due to the reduced particle size after grinding. The increased specific surface area of S3 contributes to the high adsorption capacity of MB molecules compared with the other two subdivisions. Besides, the Zeta potential of lignin particles, which is critical for the adsorption based on the electrostatic attraction, was also determined (Table 1). All lignin particles exhibited negative charges on their surface owing to the deprotonation of acidic groups (e.g., phenolic OH and COOH) in lignin molecule. Among the three lignin subdivisions, S3 had the highest negative charge (−35.24 mv) on its surface followed by S2 (−28.69 mv) while S1 had the lowest negative charge (−19.31 mv). These results can be explained by the larger specific surface area of S3, which results in more active sites (deprotonated groups) and higher negative charges [37,38]. The large negative charge of S3 increases the electrostatic attraction of cationic MB on the particle surface and the MB adsorption capacity.

### 2.2. Adsorption Capacities of Lignin Subdivisions

**Figure 4:**
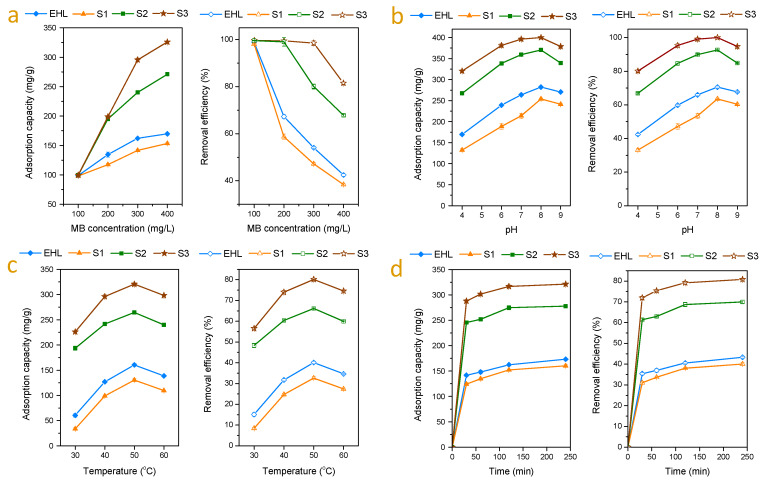
Methylene blue (MB) adsorption on EHL and its three subdivisions: (**a**) effect of MB concentration on adsorption capacity and removal efficiency (lignin concentration 1000 mg/L, temperature 50 °C, time 120 min, and pH value 4.0); (**b**) effect of pH (lignin concentration 1000 mg/L, MB concentration 400 mg/mL, temperature 50 °C and time 120 min); (**c**) effect of temperature (lignin concentration 1000 mg/L, MB concentration 400 mg/L, time 120 min, and pH value 4.0); (**d**) effect of time (lignin concentration 1000 mg/L, MB concentration 400 mg/L, pH value 4.0, and temperature 50 °C).

Batch experiments were performed in order to assess the performance of the three lignin subdivisions as absorbents for MB removal from simulated wastewater. Figure 4a shows the effect of initial MB concentration on the adsorption ability of the three lignin samples as well as the parent EHL. At low MB concentrations (100 mg/L), all lignin samples could adsorb MB completely, resulting in the same adsorption capacity (about 100 mg/g) and almost 100% removal efficiency. When the MB concentration was increased to 200 mg/L, S1 showed the lowest MB adsorption capacity (117.39 mg/g) among the three lignin subdivisions. Besides, the MB adsorption capacities of S2 and S3 were almost the same since both of them removed all MB in the solution. Once the MB concentration further increased to 300 mg/L, only S3 was able to realize the complete removal of MB from the aqueous solution while the adsorption capacity of S2 was 240.13 mg/g. These results indicate that the three lignin subdivisions show different MB adsorption capacities: S3 has the highest adsorption capacity, followed by S2, while S1 has the lowest adsorption capacity. This result is possibly caused by the high specific surface area and high Zeta potential of S3 particles, as presented in Section 2.1. The further increase of MB concentration up to 400 mg/L and the adsorption capacities of all the three lignin samples increased due to the enhanced mass transfer driving force [15]. At this concentration (400 mg/L), the MB removal efficiency of S3 was 81.46%, suggesting the achievement of saturation adsorption. Thus, MB concentration of 400 mg/L was chosen for further adsorption experiments in this study in order to avoid the insufficient adsorption of MB.

Generally, the solution pH is critical to the charge distribution of the adsorbent and the adsorbate, which determines the electrostatic or molecular interactions between the absorbent and the adsorbate and accordingly, the adsorption capacity [35,39]. The effect of solution pH on the MB adsorption capacity of the lignin samples is presented in Figure 4b. It was found that the solution pH had a considerable impact on MB adsorption of the lignin samples. The MB adsorption capacity of lignin samples increased distinctly with the increasing of the pH value from 4.0 to 8.0, while decreased with the further rise of the pH to 9.0. These results can be explained by the fact that with the increasing of the solution pH from 4.0 to 8.0, more phenolic OH and COOH groups are deprotonated [23]. Thus, the negative charge on the surface of lignin particles increases and, therefore, the MB adsorption amount enhances. However, the further increase of pH to 9.0 may result in the partial dissolution of lignin, which reduces the adsorption capacity of the lignin sample.

It was also found from Figure 4b that S3 exhibited the highest adsorption capacity and MB removal efficiency among the three subdivisions at all pH values. The effect of adsorption temperature and contacting time on the MB adsorption capacity of EHL and its three subdivisions were also investigated (Figure 4c,d). Elevating the temperature (30 °C to 50 °C) and prolonging the adsorption time (0.5 to 4 h) were helpful to increase MB adsorption capacity of lignin samples. As expected, under all conditions in Figure 4c,d, S3 exhibited the highest adsorption capacity and MB removal efficiency among the three investigated adsorbents. After optimization of the adsorption conditions (lignin concentration 1000 mg/L, MB concentration 400 mg/L, pH 8.0, temperature 50 °C and time 120 min), the maximum adsorption capacity and removal efficiency of S3 were found to be 396.85 mg/g and 99.21%, respectively. At this condition, the adsorption capacity of S3 increased about 41% compared with the parent EHL (adsorption capacity 281.54 mg/g), which means that the simple fractionation process of EHL is an efficient route to obtain a lignin subdivision with a significantly enhanced MB adsorption capacity.

The surface morphologies of S3 particles and MB adsorbed S3 particles were compared (shown in Figure S1). It was found that the MB-loaded S3 particles exhibited bigger particle size compared with the original S3 particles. The morphological change of S3 particles after adsorbing MB is possibly due to the aggregation of S3 particles. During the adsorption, the cationic MB interacts with the negatively charged S3 particles and neutralizes the charge on the particle surface, which results in the aggregation of lignin particles. The EDS chemical analyses of S3 before and after MB adsorption were also analyzed and shown in Figure S1. The trace Na and Cl detected from the EDS of S3 possibly originated from the lignin isolation process that used NaOH solution to extract lignin from EHR and precipitates lignin by the addition of HCl. The EDS spectra of MB-adsorbed S3 showed the existence of N and S elements, which clearly demonstrates the adsorption of MB by the lignin particles. In order to confirm the adsorption of MB by other lignin samples, their EDS spectra were also recorded (Figure S2) and the element compositions based on the EDS spectra after normalization were tabulated as seen in Table S1. The highest contents of N and S elements on the surface of S3 particles further confirmed the best adsorption performance of S3.

### 2.3. Adsorption Kinetics and Isotherms Studies

**Figure 5:**
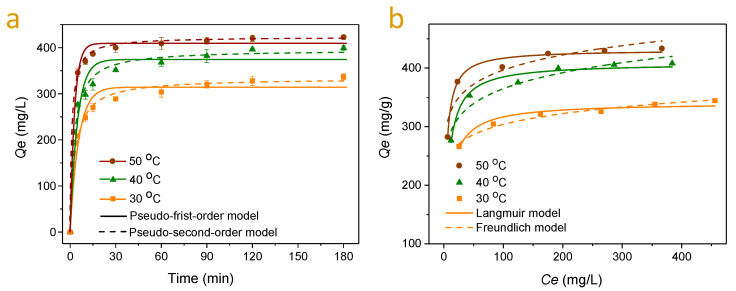
(**a**) Adsorption kinetics of MB on S3 (MB: 400 mg/L, 120 mL; adsorbent dose: 0.1 g; pH: 8) and the non-linear fitting curves using pseudo-first-order and pseudo-second-order models. (**b**) Adsorption isotherms of MB on S3 (adsorbent dose: 1 mg/mL, 10 mL; pH: 8; contact time 120 min) and the non-linear fitting curves using Freundlich and Langmuir models.

The adsorption kinetics of MB on S3 at different temperatures are presented in Figure 5a. The adsorption amount enhanced sharply at the first 10 min, then increased slowly and later tended to stabilize after 120 min. The kinetic coefficients of pseudo-first-order and pseudo-second-order models are listed in Table 2. It was observed that the calculated equilibrium adsorption capacities (*Qe*) from pseudo-second-order kinetics were closer to the experimental values at different temperatures. Moreover, *R*^2^ values obtained from the pseudo-second-order model were higher compared with those from the pseudo-first-order model. These results suggested that the MB adsorption on S3 took place according to the pseudo-second-order model, which agreed with the previous studies [22,23]. The overall rate of adsorption process is controlled by the chemical adsorption involving charge interaction between MB and S3 [23].

The MB adsorption isotherms on S3 at various temperatures are shown in Figure 5b. The equilibrium adsorption amount *Q_e_* enhanced with the increasing of MB equilibrium concentration *C_e_* in solution. The curves of the adsorption isotherms fitted by Freundlich and Langmuir models are also presented in Figure 5b and the fitting parameters of the Freundlich and Langmuir equations are listed in Table 2. By comparing the correlation coefficients (*R*^2^), it was found that the adsorption was better described by the Langmuir model compared to the Freundlich model. Besides, the Langmuir constant values of *Q_m_* matched very well to the experimental values (Table 2). Therefore, these results suggested that the adsorption of MB on S3 was a monolayer adsorption. Zhang et al. obtained similar results when they used organosolv lignin from rice straw to remove MB from aqueous solutions [23]. The maximum monolayer adsorption capacity of S3 was found to be 431.1 mg/g, which was much higher than those of most bio-absorbents from lignin and lignin-contained biomass (Table 3). Moreover, it is notable that the MB adsorption capacity of S3 is comparable to those of biomass derived activated carbons (Table 3). Thus, the result indicates that the S3 obtained through the simple fractionation approach has a similar MB adsorption capacity as the biomass derived activated carbons prepared using a thermochemical modification process [40,41].

### 2.4. Recycling Studies

**Figure 6:**
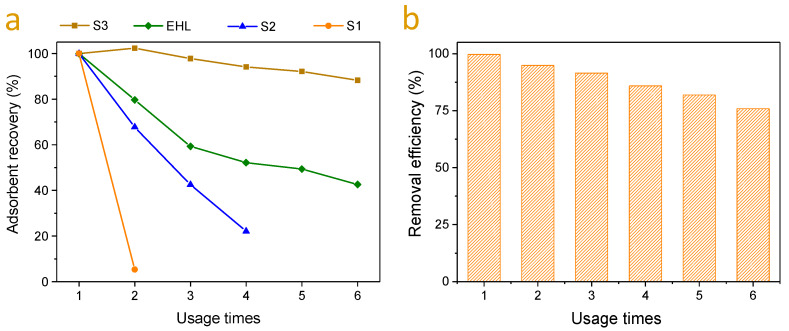
(**a**) Recovery of lignin adsorbents (S1, S2, S3, and EHL) under different recycle times; (**b**) adsorption capacities of S3 under different recycle times.

To evaluate the adsorbent recyclability, the used lignin adsorbents including the EHL and its three subdivisions, were regenerated by washing with ethanol and then reused to adsorb MB. The recoveries of the four lignin adsorbents are presented in Figure 6a. The S1 recovery after ethanol washing was only 5.36%. This was because S1 is the soluble fraction of EHL in 95% ethanol, and after ethanol washing, most of S1 dissolves. Similarly, S2 also exhibited a low recovery since it was soluble in 80% ethanol. Compared with S1 and S2, S3 had much higher recovery (over 90% after 5 recycles) due to its insolubility in both 95% and 80% ethanol solutions. The recovered S3 was then reused for up to six operation cycles and the corresponding MB adsorption capacities are shown in Figure 6b. Although the adsorption capacity of regenerated S3 was slightly decreased, it still exhibited over 75% of the original adsorption capacity after 5 cycles of regeneration. These results indicate that S3 exhibits satisfactory recycling performance via ethanol washing regeneration, showing excellent potential in practical applications.

## 3. Materials and Methods

### 3.1. Material

Enzymatic hydrolysis residue (EHR) was kindly supplied by Songyuan Laihe Chemical Co. Ltd. (Jilin, China). The enzymatic hydrolysis (cellulase loading 30 U/g, temperature 50 °C and time 48 h) was carried out using steam exploded corn stalk (steam pressure 1.5 MPa and treatment time 5 min) as the raw material [28]. All laboratory reagents used in this work were analytically pure.

### 3.2. Isolation of Enzymatic Hydrolysis Lignin from Enzymatic Hydrolysis Residue

The isolation of EHL from EHR was based on alkaline extraction followed by acid precipitation [28]. Specifically, 20.0 g of EHR was added to 400 mL of 1% NaOH (*w*/*v*) solution and then the mixture was heated at 80 °C for 2 h with mechanical stirring (120 rpm). After alkaline extraction, the liquid and solid fractions were separated by a centrifugation at 5000 rpm for 10 min. The lignin extracted liquor (supernatant) was obtained and then adjusted to pH 2.0 using diluted HCl (1 mol/L) with vigorous agitation at ambient temperature. The precipitated EHL was then obtained by centrifugation (6000 rpm, 5 min) and washed three times with 400 mL distilled water. Afterward, the cleaned EHL was dried at 55 °C for 12 h using a vacuum oven (VOS-30A, STIK, Shanghai, China) and ground by a mortar to pass through a 120-mesh sieve.

### 3.3. Fractionation of Enzymatic Hydrolysis Lignin

The EHL was subdivided by stepwise dissolution in 95% and 80% ethanol solvents to obtain three lignin subdivisions according to the previous study [25]. Briefly, 5.0 g of EHL was added into 250 mL of 95% ethanol solution (ethanol/ethanol+water, *v*/*v*) and the mixture was stirred mechanically at 200 rpm for 10 min at ambient temperature. Afterward, the mixture was centrifuged at 6000 rpm for 10 min to obtain the supernatant (S1) and the insoluble lignin. The insoluble lignin was subsequently added in 250 mL of 80% ethanol solution (ethanol/ethanol+water, *v*/*v*). After stirring at 200 rpm for 10 min, the mixture was centrifuged at 5000 rpm for 5 min to obtain the supernatant (S2) and the insoluble lignin subdivision (S3). The S1 and S2, dissolved in 95% and 80% ethanol solutions, respectively, were recovered by vacuum rotary evaporation. All three subdivisions were further dried in a vacuum oven at 55 °C for 12 h and ground by a mortar to pass through a 120-mesh sieve.

### 3.4. Characterization of EHL and Lignin Subdivisions

Molecular weight distribution was measured by gel permeation chromatography (GPC) using a hydrophilic gel column (TSK G3000PWxl column, Tosoh Co., Tokyo, Japan).) [25]. The lignin samples were dissolved into 1% NaOH solution and diluted in tris-acetate buffer (20 mmol/L, pH 7.4). The column was operated at 25 °C and eluted with tris-acetate buffer (20 mmol/L, pH 7.4) at a flow rate of 0.5 mL/min. FTIR spectra were acquired using the KBr technique through a FTIR spectrophotometer (FTIR-650, Gangdong Sci. & Tech. Tianjin, China) [28]. The samples were firstly mixed with KBr by a ratio of 1:100 (sample to KBr, *w*/*w*), and the mixture was then ground in an agate mortar and compressed to obtain the KBr disc for FTIR determination. The wavenumber region was between 4000 and 400 cm^−1^ with a resolution of 4 cm^−1^ and 20 scans were recorded. Zeta potential and particle size distribution of lignin samples were analyzed by dynamic light scattering measurement (Malvern Instruments Ltd., Malvern, UK). Here, lignin solutions were prepared with an identical concentration and adjusted to the same pH value for Zeta potential and particle size determination. The specific surface area of lignin samples was measured through N_2_ adsorption using a specific surface area analysis instrument (Quantachrome Autosorb-IQ, Boynton, FL, USA) at a liquid nitrogen temperature [43]. The morphology of lignin adsorbents was observed using Scanning electron microscopy (SEM) measurements (SHIMADZU SSX-550, Kyoto, Japan), combined with Energy-dispersive X-ray spectroscope (EDS) for the determination of chemical compositions of lignin adsorbents before and after the adsorption. Before the analysis, a small amount of lignin samples was evenly spread on the conductive adhesive and a thin gold coating was deposited onto the uniformly.

### 3.5. Batch experiments for Methylene Blue Adsorption

MB Adsorption experiments from aqueous solutions by the lignin samples (S1, S2, S3, and EHL) were carried out in triplicate using 50 mL Erlenmeyer flasks containing 10 mL of MB solution with different initial concentrations (C_0_, 100–400 mg/L). The pH of the MB solution ranging from 2.0 to 9.0 was adjusted by adding 0.1 M NaOH and 0.1 M HCl solutions. Lignin sample with a fixed amount was added into the MB solution and the mixture was shaken in a thermostatic shaking incubator (120 rpm) at predetermined temperatures. After adsorption, the mixture was centrifuged at 5000 rpm for 5 min to collect the lignin adsorbent from the MB aqueous solution. The residual MB in the solution was measured using a UV-visible Spectrophotometer (Shimadzu UV-2500, 664 nm, Kyoto, Japan) [43]. The adsorption capacity (*Q*_t_) and percentage of MB removal (*E*) were obtained according to Equations (1) and (2), respectively [47]:

(1) $$Q_{t}\left(\mathrm{mg}/g\right)=\frac{(C_{0}-C_{t})V}{m}$$

(2) $$E(\%)=\frac{\left(C_{0}-C_{t}\right)}{C_{0}}\times 100$$

where *C*_0_ is the initial MB concentration (mg/L) and *C*_t_ is the MB concentration in mg/L at time *t* (min), *m* is the weight of lignin adsorbent in g, and *V* is the volume of the MB solution in L.

### 3.6. Adsorption Kinetics and Isotherms

The kinetics of the MB adsorption process by the 80% ethanol insoluble subdivision (S3) was studied by contacting 120 mL 400 mg/L MB solution (pH 8.0) with 100 mg lignin at 30, 40 and 50 °C. The pseudo-first and second-order models were employed to simulate the adsorption kinetics and the equations were represented as the following [48]:

Pseudo-first-order models:

(3) $$Q_{t}=Q_{e}(1-\exp^{-k1t})$$

Pseudo-second-order models:

(4) $$Q_{t}=\frac{k_{2}{Q_{e}}^{2}t}{1+k_{2}Q_{e}t}$$

where *Q*_t_ (mg/g) is the amount of MB adsorbed at time *t* (min), *k*_1_ and *k*_2_ are the rate constants of pseudo-first-order and pseudo-second-order models, respectively.

The isotherms of MB adsorption on lignin were investigated by contacting 10 mL MB solution (pH 8.0, 300–800 mg/L) with 10 mg lignin at 30, 40 and 50 °C for 120 min. Freundlich and Langmuir isotherm models were used to simulate the adsorption data [49]:

Freundlich model:

(5) $$Q_{e}=k_{F}{C_{e}}^{1/n}$$

Langmuir model:

(6) $$Q_{e}=\frac{Q_{m}k_{L}C_{e}}{1+k_{L}C_{e}}$$

where *Q_e_* is the equilibrium MB adsorption amount of lignin (mg/g), *C_e_* is the equilibrium MB concentration (mg/L), and *Q_m_* (mg/g) is the monolayer maximum adsorption ability. In the Freundlich model, *n* and *k_F_* represent the intensity of adsorption and the multilayer adsorption capacity (mg/g), while *k_L_* in Langmuir model is the Langmuir constant (L/mg).

### 3.7. Lignin Recycling

Adsorbent recyclability was also studied by regeneration of MB loaded adsorbent in ethanol solution [50,51]. Typically, 0.01 g lignin was added into a 50 mL Erlenmeyer flask with 10 mL of 400 mg/mL MB solution. The pH of the MB solution was 8.0. The mixture was shaken in a thermostatic shaking incubator (120 rpm) at 50 °C for 180 min. Once the adsorption equilibrium was achieved, the used lignin was collected by centrifugation (5000 rpm, 5 min). The MB desorption from the lignin adsorbents was conducted by ultrasonically washing with 10 mL ethanol for 4 times. Finally, the lignin adsorbent was washed with distilled water and ground to pass through a 120-mesh sieve after vacuum drying. The regenerated lignin was used for the next adsorption run following the identical experimental conditions. The adsorption-desorption process was repeated at least 6 times using the same lignin adsorbent.

## 4. Conclusions

Three lignin subdivisions with decreased heterogeneity of molecular weight and structure were obtained by stepwise dissolution of EHL in 95% and 80% ethanol solutions. The 80% ethanol-insoluble subdivision (S3) showed the highest adsorption capacity (396.85 mg/g). The dissolution of S1 and S2 resulted in the decrease of S3 particle size and the formation of a rough surface, which led to the increase of specific surface area (4.84 m^2^/g) and the negative Zeta potential (−35.24 mv), and, therefore, the adsorption capacity. The MB adsorption on S3 followed pseudo-second-order kinetics and based on the adsorption isotherms fitted by Langmuir model, the maximum monolayer adsorption capacity of MB on S3 was 431.1 mg/g, which was much higher than those of most reported lignin-based adsorbents. Moreover, S3 demonstrated much better recyclability owing to its high regeneration yield (over 90% after 5 recycles) by ethanol desorption. Overall, the adsorption capacity and recyclability of EHL was significantly improved using the simple stepwise ethanol-water fractionation process.

## Figures and Tables

**Table 1:** Yield, molecular weight distribution, specific surface area and Zeta potential of lignin subdivisions, as well as those of enzymatic hydrolysis lignin (EHL).

	S1	S2	S3	EHL (S1 + S2 + S3)
Yield (%)	22.87	27.80	45.34	-
M_w_	4050	6270	12230	7280
M_n_	2238	3800	7194	3569
Polydispersity	1.81	1.65	1.70	2.04
Specific surface area	2.44	4.06	4.84	2.89
Zeta potential ^1^	−19.31 ± 1.63	−28.69 ± 2.29	−35.24 ± 3.08	−24.71 ± 1.77

^1^ Detected at pH 6.0.

**Table 2:** Kinetic and isotherm parameters for adsorption of methylene blue on 80% ethanol insoluble subdivision (S3).

**Kinetic parameters**	***T* (°C)**	***Q_e_* (mg/g)**	**Pseudo-First-Order Kinetics**			**Pseudo-Second-Order Kinetics**		
			***K_1_* (min^−1^)**	***Q_1_* (mg/g)**	***R*^2^**	K_2_*10^−4^ (g_*_mg^−1^_*_min^−1^)	***Q_2_* (mg/g)**	***R*^2^**
	30	337.7	0.18	312.5	0.966	9.3	331.1	0.994
	40	395.1	0.21	372.0	0.952	9.7	392.5	0.989
	50	418.9	0.35	406.9	0.987	20.2	420.1	0.999
**Isotherm parameters**	***T* (°C)**		**Freundlich Constant**			**Langmuir Constant**		
			***n***	***K_F_***	***R*^2^**	***b* (L/mg)**	***Q_m_* (mg/g)**	***R*^2^**
	30		11.8	205.4	0.972	0.13	341.0	0.931
	40		9.5	225.6	0.916	0.16	408.5	0.969
	50		11.0	260.8	0.858	0.29	431.1	0.979

**Table 3:** The maximum monolayer adsorption (*Q_m_*) of methylene blue onto various adsorbents.

Adsorbents	Adsorption Capacity (mg/g)	References
80% insoluble subdivision of EHL (corn stalk)	431.1	This work
Deacetylated acetic acid lignin (eucalyptus)	63.3	[22]
Organosolv lignin (rice straw)	40.0	[23]
Formic lignin (sugar cane bagasse)	34.2	[42]
Fe_3_O_4_@lignosulfonate/phenolic microsphere	292.6	[43]
Straw based adsorbents	274.7	[44]
Swede rape straw	246.4	[45]
Bamboo-based activated carbon	454.2	[46]
cork waste-based activated carbon	350.0	[40]
Coconut husk-based activated carbon	434.8	[41]

## Supplementary Materials

The following are available online, Figure S1: SEM (a and c) and EDS analyses (b and d) of S3 (a and b) and MB adsorbed S3 (c and d), Figure S2: EDS analyses of lignin before and after methylene blue adsorption, Table S1: Element compositions of lignin and MB adsorbed lignin based on EDS spectra after normalization.
