# Supplementary material for: Stepwise Ethanol-Water Fractionation of Enzymatic Hydrolysis Lignin to Improve Its Performance as a Cationic Dye Adsorbent
Source: Molecules. 2020 Jun 3;25(11):2603. doi: 10.3390/molecules25112603 (PMC7321336; doi:10.3390/molecules25112603)
Supplement: Supplementary file 1 [file molecules-25-02603-s001.pdf]

Supplementary Materials for:

Stepwise Ethanol-Water Fractionation of Enzymatic Hydrolysis Lignin to Improve Its Performance as a Cationic Dye Adsorbent

Wenjie Sui [1,*,†], Tairan Pang [2,†], Guanhua Wang [2,3,*], Cuiyun Liu [2], Ashak Mahmud Parvez [4], Chuanling Si [2] and Chao Li [3,5]

1 State Key Laboratory of Food Nutrition and Safety, College of Food Science and Engineering, Tianjin University of Science & Technology, Tianjin 300457, China
2 Tianjin Key Laboratory of Pulp and Paper, College of Light Industry Science and Engineering, Tianjin University of Science and Technology, Tianjin 300457, China; pangtr1995@163.com (T.P.); zclcy1214@163.com (C.L.); sichli@tust.edu.cn (C.S.)
3 Hunan BISEN Environmental & Energy Co., Ltd., Changsha 410100, China; chaoli_tu@tsinghua.edu.cn
4 Department of Mechanical Engineering, University of New Brunswick, Fredericton, NB E3B 5A3, Canada; Ashak.Parvez@unb.ca
5 School of Environment, Tsinghua University, Beijing 100084, China
* Correspondence: wjsui@tust.edu.cn (W.S.); ghwang@tust.edu.cn (G.W.); Tel.: +86-02260601313 (G.W.)
† These authors contributed equally to the work

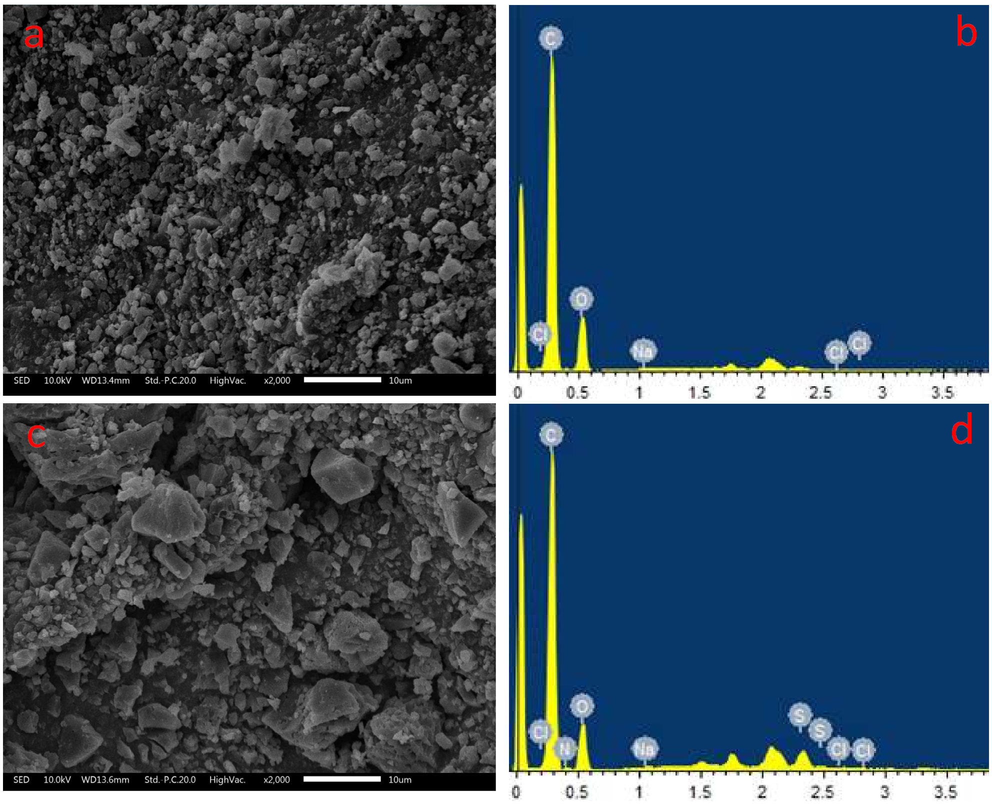

Figure S1. SEM (a and c) and EDS analyses (b and d) of S3 (a and b) and MB adsorbed S3 (c and d).

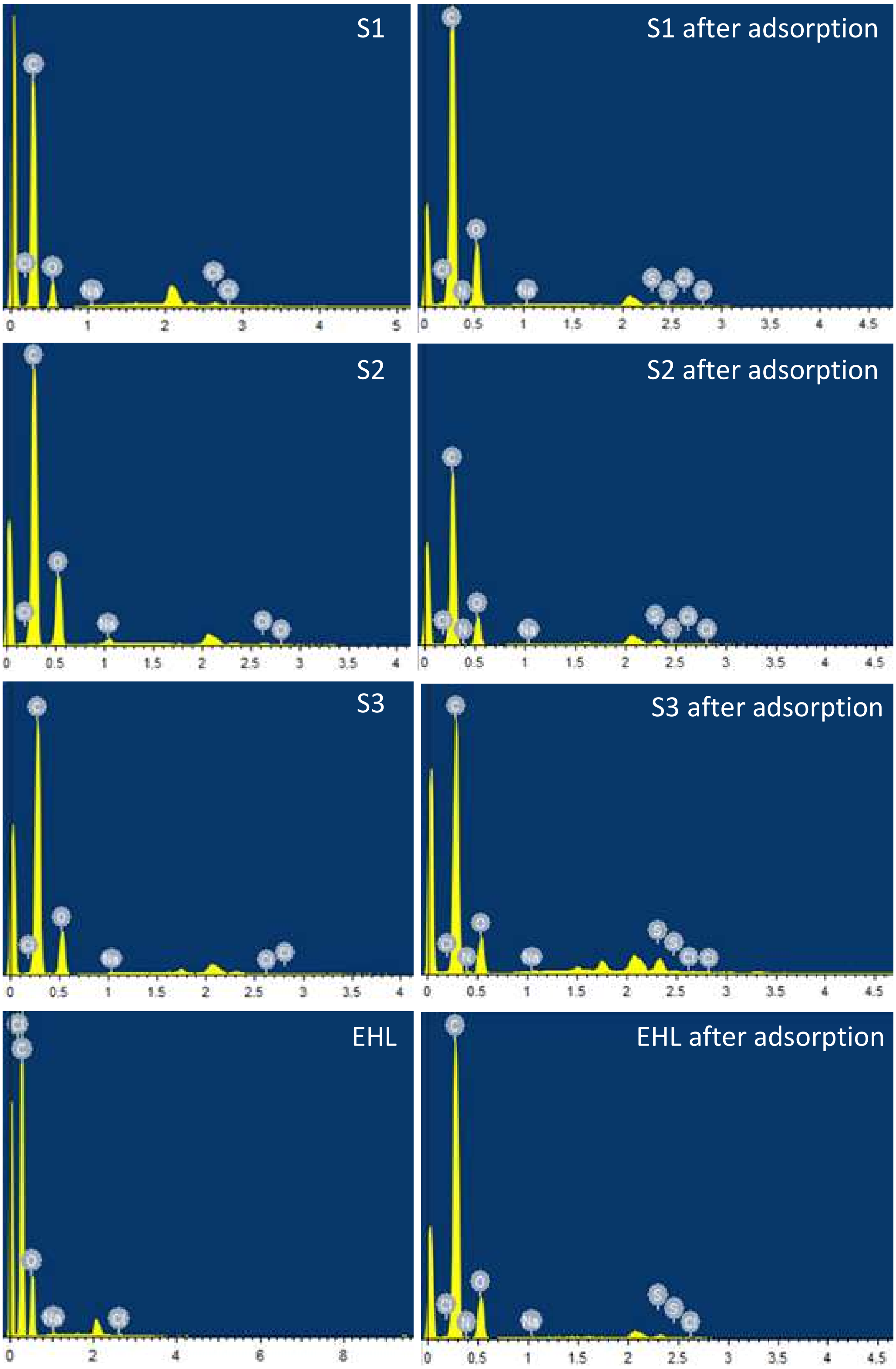

Figure S2. EDS analyses of lignin before and after methylene blue adsorption.

Table S1. Element compositions of lignin and MB adsorbed lignin based on EDS spectra after normalization.

Sample	C	O	Na	Cl	N	S
S1	63.19±1.73	35.31±1.87	0.12±0.04	0.38±0.14	-	-
S2	63.62±1.61	35.58±1.79	0.67±0.06	0.27±0.12	-	-
S3	67.83±1.05	32.09±1.08	0.07±0.01	0.02±0.01	-	-
EHL	67.92±1.87	31.71±1.87	0.20±0.01	0.18±0.02	-	-
S1 after adsorption	67.99±2.80	30.92±2.51	0.04±0.01	0.02±0.01	0.74±0.27	0.33±0.14
S2 after adsorption	68.30±1.27	29.09±0.16	0.05±0.03	0.04±0.01	2.03±0.16	0.52±0.24
S3 after adsorption	69.84±4.86	24.15±2.74	0.07±0.02	0.06±0.02	4.40±1.06	1.48±0.02
EHL after adsorption	69.73±1.94	27.64±2.32	0.04±0.01	0.02±0.01	1.90±0.35	0.67±0.16
